# Is the association between working from home and higher frequency of drinking and heavy episodic drinking causal? A longitudinal analysis in the Norwegian workforce

**DOI:** 10.5271/sjweh.4217

**Published:** 2025-05-01

**Authors:** Torleif Halkjelsvik, Inger Synnøve Moan

**Affiliations:** 1 Department of Alcohol, Tobacco, and Drugs, Norwegian Institute of Public Health, Norway.

**Keywords:** alcohol consumption, remote work

## Abstract

**Objectives:**

There have been concerns that the shift to more home-based work might result in increased alcohol consumption due to reduced supervision and increased accessibility of alcohol. Empirical studies indicate associations between working from home and alcohol consumption. We go beyond cross-sectional associations by using longitudinal data and directly inquiring about alcohol consumption while working from home.

**Methods:**

Based on demographics of the Norwegian workforce, participants were recruited from an online research panel (sample sizes N=1257–4294) before (2018–2019), during (2020–2021) and after (2022–2023) pandemic restrictions that encouraged or mandated remote work. Fixed effects regression analyses controlled for stable individual-level characteristics.

**Results:**

On average, employees working from home reported 28% more drinking episodes and 26% more heavy episodic drinking (HED) compared to other employees. However, changes in the frequency of remote workdays were not notably related to the frequency of drinking [B=0.02, 95% confidence interval (CI) -0.12–0.16] or HED (B=0.05, 95% CI -0.08–0.19). Furthermore, relative to other employees, employees working from home during the pandemic restrictions in 2020 and 2021 did not increase their drinking or HED frequency from pre-pandemic levels (B= -0.28, 95% CI -0.74–0.18 and B=0.02, 95%CI -0.21–0.24, respectively). Few workers reported weekly alcohol consumption during office hours while working from home (1%).

**Conclusions:**

The cross-sectional relation between working from home and alcohol consumption found in past studies was replicated, but, using longitudinal data, we demonstrated that employee characteristics confound the relation. Our findings indicate that alcohol consumption during home-based work is unlikely to constitute a significant public health threat.

Early in the COVID-19 pandemic, scholars warned that long-term isolation due to pandemic-related measures could create a public health crisis involving increased alcohol consumption (1). One important change in occupational life was the requirement or encouragement of remote work for those able to perform their tasks from home. This change entails less social contact and thereby reduced social control, a factor associated with increased alcohol consumption among employees (2).

Alcohol consumption patterns changed after the onset of the pandemic. In most countries, the tendency was to report a reduction in alcohol consumption (3). However, some individuals reduced their consumption whereas others increased theirs, depending on socio-demographic characteristics and alcohol consumption levels (4–6). The changes in individuals’ drinking habits during the pandemic might be related to working conditions, such as the increased use of home office. Of workers who reported an increase in alcohol consumption during the initial months of the pandemic, one in three attributed the changes to a more flexible work situation (6). Studies conducted in Norway, Ireland and Poland showed that an increase in alcohol consumption was more prevalent among those working from home (7–9). In contrast, a Japanese study found that working from home was associated with decreased alcohol consumption (10). Two Norwegian studies have reported associations between working from home and higher alcohol consumption (11, 12).

The aforementioned studies suggest that working from home may increase drinking. However, this link has typically been explored cross-sectionally and may therefore reflect variation in alcohol consumption between different sociodemographic groups of employees. The current study uses data collected over time, as well as direct inquiries about alcohol consumption while working from home, to circumvent the possible confounding effects of socio-demographics. We first demonstrate a cross-sectional association between working from home and alcohol consumption, then our aim is to explore three research questions that further illuminate this relation: (i) Are changes in the number of days working from home associated with changes in drinking? (ii) Did employees who worked from home during the pandemic restrictions increase their alcohol use more than other employees? and (iii) How many, when asked directly, report that they drink while working from home?

## Methods

### Participants

Data are from a larger data collection initiated in 2015, with samples drawn annually from Kantar’s research panel, based on characteristics of the Norwegian working population. Approximately 60% of each sample was drawn from respondents of the previous surveys, while 40% were new participants. We used three different samples derived from the larger survey: a pooled sample comprising all data from years 2020–2023 (see supplementary materials, www.sjweh.fi/article/4217, table S1, upper panel) used in the illustrative cross-sectional analysis, one longitudinal sample comprising those who participated more than once during 2020–2023 (see supplementary table S1, middle panel), and another longitudinal sample including all individuals who participated at least once before (2018–2019) and after (2020–2023) the onset of the pandemic (supplementary table S1, lower panel).

### Measures

Alcohol consumption frequency was measured by asking “During the past 12 months, approximately how often have you drunk alcohol?”. Heavy episodic drinking (HED) was measured with the question: “During the past 12 months, approximately how often have you drunk [≥6 for men and ≥4 for women] alcohol units on a single occasion?”. We calculated drinking episodes per month (see supplementary tables S2–S3).

Working from home was measured in 2020–2023: “Approximately how many days per week do you have home office this month?” (supplementary table S4). The direct question about drinking while working from home was “Do you ever drink alcohol during regular working hours when you have home office?”

### Analysis

The first longitudinal analysis used data from 2020–2023, regressed drinking frequency/HED on number of days working from home, and included fixed year and person effects (see supplementary text S1). The second longitudinal analysis used data from 2018–2023, regressed drinking frequency/HED on two dummy variables for the periods during (2020–2021) and after (2022–2023) pandemic-related restrictions, and included fixed person effects. Interaction terms between the dummy variables and a “treatment” indicator (working from home) estimated the differences between those who could and could not work from home in their changes from pre-pandemic levels to the periods during and after restrictions. Employees working from home ≥1 day per week in both 2020 and 2021 (while pandemic-related restrictions were in force) were the ‘treatment’ group, and employees not working from home the comparison group. The analysis relies on the assumption that remote work was substantially less common before the onset of the pandemic (13).

## Results

### Cross-sectional analyses

To illustrate the cross-sectional association reported in past studies, we compared the group who reported that they worked from home with those who did not work from home. Across all observations, the average drinking frequency per month was 4.22 [standard deviation (SD) 5.01] for employees not working from home and 28% higher (mean 5.41, SD 5.64) for employees working from home one or more days. The average number of HED occasions per month was 1.32 (SD 2.69) for employees not working from home and 26% higher (mean 1.66, SD 3.33) for those working from home. Welch t-tests of differences in means, each year 2020–2023, suggested higher frequency of drinking and more HED occasions among employees who worked from home (P<0.05 for both measures, all years, except HED in 2023).

### Longitudinal analyses

In the first longitudinal analysis, we assessed the within-person variability in frequency of drinking as a function of the within-person variability in number of days working from home during 2020–2023. The analysis did not detect any association between home office and drinking frequency [B=0.02, t=0.33, P=0.74, 95% confidence interval (CI) -0.12–0.16 (observations=2851, N=1248)]. A corresponding analysis of HED gave similar results (B=0.05, t=0.81, P=0.42, 95% CI -0.08–0.19).

In the second longitudinal analysis, meant to supplement the first using a different approach, we assessed the within-person changes from before (2018–2019) to during (2020–2021) and after (2022–2023) pandemic restrictions were in effect. In analyses of drinking frequency, we detected no changes from pre-pandemic levels and no differences in changes according to working from home status (table 1, upper panel). In corresponding analyses of HED (table 1, lower panel), the group not working from home showed an increase of about 0.25 episodes per month during the pandemic restrictions, which also persisted after the restrictions. Analyses did not suggest that the increase was different for those who could work from home.

**Table 1:** Development in drinking frequency and heavy episodic drinking (HED) per month during and after pandemic-related restrictions interacted by working from home (WFH) during restrictions (no days versus one or more days per week). [CI=confidence interval; Obs=observations.] ^a^

		B	t	P-value	95% CI
Frequency ^b^					
	Before (2018/2019)	Reference			
	During (2020/2021)	0.04	0.41	0.68	-0.17–0.26
	After (2022/2023)	-0.06	-0.32	0.75	-0.40–0.29
	During by WFH	-0.28	-1.20	0.23	-0.74–0.18
	After by WFH	-0.52	-1.52	0.13	-1.20–0.15
HED ^c^					
	Before (2018/2019)	Reference			
	During (2020/2021)	0.23	3.72	<0.001	0.11–0.35
	After (2022/2023)	0.25	2.35	0.019	0.04–0.46
	During by WFH	0.02	0.15	0.88	-0.21–0.24
	After by WFH	-0.07	-0.33	0.74	-0.46–0.33

^a^ Cluster-robust standard errors were grouped on individuals (N=1632 and N=1562). WFH is coded 0 (no days working from home) and 1 (≥1 days per week). The main effect of WFH is redundant due to fixed effects of individuals. ^b^ Obervations=5643. ^c^ Obervations=5326.

### Direct question about alcohol consumption while working from home

Of employees who worked from home, 3.7% (95% CI 2.7–4.6) reported drinking alcohol weekly or more often during office hours when working from home (see table 2). In the pooled sample, including workers who did not work from home, this corresponds to 1.1% (95% CI 0.8–1.4).

**Table 2:** The frequency of drinking while working from home the previous month.

Times per week	2020			2021			2022			2023			Total	
	N	%		N	%		N	%		N	%		N	%
Never	416	92.9		465	94.9		415	93.1		355	93.2		1651	93.5
<1	12	2.7		11	2.2		15	3.4		11	2.9		49	2.8
1	6	1.3		3	0.6		6	1.4		7	1.8		22	1.3
2	9	2.0		6	1.2		9	2.0		7	1.8		31	1.8
3	3	0.7		3	0.6		0	0.0		0	0.0		6	0.3
4	0	0.0		0	0.0		1	0.2		0	0.0		1	0.1
≥5	2	0.5		2	0.4		0	0.0		1	0.3		5	0.3
Total	448	100		490	100		446	100		381	100		1765	100
No home office	1022	69.0		991	66.6		1049	70.0		1069	73.6		4131	69.8
Missing	12	0.7		8	0.4		4	0.2		4	0.2		24	0.4

## Discussion

Our cross-sectional analyses of alcohol consumption among Norwegian workers revealed the pattern observed in past studies—working from home is associated with more drinking. However, the association between within-person changes in the extent of remote work and changes in alcohol consumption was estimated as close to zero. The pandemic led to a sudden increase in remote work, and when considering its onset as a naturally occurring shock, our analyses did not detect differences in changes from pre-pandemic alcohol consumption levels between those who could and could not work from home. Few workers reported consuming alcohol while working from home.

In past studies, an increase in alcohol consumption during the pandemic was more common among those working from home (7–9). The present conclusions diverge from these studies. We believe this primarily reflects the difference between asking for retrospective reports of changes at one point in time and repeated measurements. The inclusion of assessments made over several years reduces the risk of confounding from stable individual characteristics, however, there is still a risk of confounding from factors that change over time. Our inclusion of direct questions about drinking while working from home circumvents this problem, however, the approach relies on the assumption that the potential increase in drinking happens during office hours. It could be the case that working from home increases drinking later in the day because one is less worried about being on time and in good shape the next day. Nevertheless, it is reassuring that the three analytical approaches, each with its own limitations, provide consistent results.

Qualitative research suggests that individuals who drink the most may increase their drinking when working from home (14). Since there are few heavy drinkers in the data, we cannot rule out such effects (see supplementary text S2). The data is from Norway, a high income, European country, which may limit generalizability. However, if increased availability of alcohol and reduced social control are the mechanisms that increase alcohol use when working from home (15), these factors should equally apply to moderate drinkers in a Norwegian context.

### Concluding remarks

Across three different analytical approaches, we find no evidence that working from home carries a general risk of increased alcohol consumption. The link between working from home and alcohol consumption, as observed in both the current and past studies, appears to be confounded by stable worker characteristics. The potential effects of working from home on alcohol consumption are likely too small to pose a threat to public health.

## Supplementary material

Supplementary material
