# Supplementary material for: Is the association between working from home and higher frequency of drinking and heavy episodic drinking causal? A longitudinal analysis in the Norwegian workforce
Source: Scand J Work Environ Health. 2025 Apr 27;51(3):255–8. doi: 10.5271/sjweh.4217 (PMC12074692; doi:10.5271/sjweh.4217)
Supplement: Supplementary material [file SJWEH-51-255-S001.pdf]

Supplemental Materials for

"Is the association between working from home and higher frequency of drinking and heavy episodic drinking causal? A longitudinal analysis in the Norwegian workforce "

Torleif Halkjelsvik[1] & Inger Synnøve Moan

Norwegian Institute of Public Health

1 Corresponding to: Torleif Halkjelsvik, Norwegian Institute of Public Health (Folkehelseinstituttet), Postboks 222 Skøyen 0213 Oslo, Norway. [E-mail: Torleif.Halkjelsvik@fhi.no]

Table S1. Sociodemographic characteristics of workers in three subsamples according to whether they work from home (WFH)

	No WFH	Any WFH	All	*Missing observations*
	Pooled subsample 2020-2023			
Individuals N	3,057[a]	1,423[a]	4,294	*22*
Observations	4,131	1,766	5,897	*23*
Women	49.9%	45.2%	48.5%	*0*
Mean age	46.8	46.2	46.6	*0*
Higher Education	39.8%	65.7%	47.6%	*23*
Income>599K	35.9%	60.1%	43.2%	*552*
Public sector	41.1%	31.2%	38.2%	*33*
Full time work	79.5%	91.1%	83.0%	*184*
Child below 15	26.5%	33.1%	28.5%	*162*
	Longitudinal subsample I (2020-2023)[b]			
Individuals N	990[a]	452[a]	1,257	*11*
Observations	2,064	795	2,859	*12*
Women	48.1%	43.7%	46.8%	*0*
Mean age	47.0	46.6	46.9	*0*
Higher Education	35.2%	61.7%	42.5%	*10*
Income>599K	34.2%	60.6%	41.6%	*281*
Public sector	39.4%	30.9%	37.1%	*14*
Full time work	79.8%	91.9%	83.2%	*78*
Child below 15	25.4%	30.6%	26.8%	*73*
	Longitudinal subsample II (2018-2023)			
Individuals N	1,100	463	1,563	*42*
Observations	3,015	1,156	4,171	*143*
Women	49.2%	45.8%	48.3%	*0*
Mean age	47.1	46.6	46.9	*0*
Higher Education	35.1%	65.9%	43.6%	*16*
Income>599K	30.3%	56.7%	37.7%	*501*
Public sector	38.9%	33.0%	37.3%	*19*
Full time work	79.2%	91.3%	82.6%	*115*
Child below 15	26.2%	31.0%	27.5%	*88*

Note. For "Individuals N" and "Observations" missing refers to WFH status. [a] "Individuals N" does not sum to "All" because individuals can be in multiple categories (including missing) depending on survey year; [b]For consistency we report sociodemographic characteristics separately according to No versus Any WFH, but the analysis reported for this subsample used any variation in number of days WFH.

Table S2. Coding of the original response scale of drinking frequency and Heavy Episodic Drinking into drinking episodes per month.

Original response option	Recoded as
Never	0
More than 3 days	0.08
About once per month	0.21
2-3 days per month	0.33
About once per week	1
2-3 days per week	2.5
About once per week	4.3
2-3 days per week	10.75
4-5 days per week	19.35
About daily	28

Table S3. Coding of original response scale of frequency of alcohol use while working from home per week into monthly drinking episodes.

Original response option	Recoded as
No, never	0
Yes, less than one day per week	1
One day per week	4.3
Two days per week	8.6
Three days per week	12.9
Four days per week	17.2
Five days per week or more	21.5

Table S4. Number of days per week working from home in percentages of sample (number of observations in parentheses)

Days	2020	2021	2022	2023	Total
None	69.48 (1,022)	66.91 (991)	70.17 (1,049)	73.72 (1,069)	70.05 (4,131)
Once	7.21 (106)	7.09 (105)	11.57 (173)	10,76 (156)	9.16 (540)
Twice	5.71 (84)	6.55 (97)	8.16 (122)	7.10 (103)	6.88 (406)
Three days	4.89 (72)	5.67 (84)	3.88 (58)	4.21 (61)	4.66 (275)
Four days	3.26 (48)	4.32 (64)	2.54 (38)	1.79 (26)	2.98 (176)
Five days+	9.45 (139)	9.45 (140)	3.68 (55)	2.41 (35)	6.26 (369)
Total	100.00 (1,471)	100.00 (1,481)	100.00 (1,495)	100.00 (1,450)	100.00 (5,897)

Note. There were 11, 6, 3, and 3 missing observations for the four years, respectively.

Supplementary Text S1. Regression Equations

The first longitudinal model (with two different dependent variables analysed separately):

$$DrinkFrequency_{it} = Individual_i + SurveyYear_t + \beta * DaysWorkingHome_{it} + \varepsilon_{it}$$

$$HED_{it} = Individual_i + SurveyYear_t + \beta * DaysWorkingHome_{it} + \varepsilon_{it}$$

where the outcome DrinkFrequency in the first equation is the number of drinking episodes per month and the outcome HED in the second equation is the number of heavy drinking episodes per month. The term Individual_i represents fixed effects of individuals, SurveyYear_t represent dummy variables for survey years 2020-2023 minus the first used as a reference category, β is the regression coefficient for the effect of the number of days working from home per week. ε_{it} is an observation-level error term. Calculation of standard errors were cluster-robust and allowed for correlated residuals within individuals.

The second longitudinal model (with two different dependent variables analysed separately):

$$DrinkFrequency_{it} = Individual_i + \beta_1 DuringRestrict_t + \beta_2 AfterRestrict_t + \beta_3 (During_t * WorkedFromHome_i) + \beta_4 (After_t * WorkedFromHome_i) + \varepsilon_{it}$$

$$HED_{it} = Individual_i + \beta_1 DuringRestrict_t + \beta_2 AfterRestrict_t + \beta_3 (During_t * WorkedFromHome_i) + \beta_4 (After_t * WorkedFromHome_i) + \varepsilon_{it}$$

where terms identical to those in the first model have the same interpretation. DuringRestrict_t and AfterRestrict_t are dummy variables (0, 1) indicating the period during restrictions in 2020-2021 and the period after restrictions in 2022-2023, with the period before (2018-2019) as the reference category.

WorkedFromHome_i is a dummy variable that takes the value 0 when the individual did not work from home during the pandemic (both years 2020-2021), and the value 1 when the individual worked from home one day or more per week both years 2020-2021. Individuals with inconsistent working from home status in 2020 and 2021 (149 participants) where excluded from this analysis.

The coefficient β_1 provides the change in the dependent variable from the baseline period 2018-2019 to the DuringRestrict period (2020-2021) for the group that did not work from home; and β_2 the change from the baseline period 2018-2019 to the AfterRestrict period (2022-2023) for the same group. The coefficient β_3 indicates the additional change from baseline to the DuringRestrict period among those who worked from home, relative to the development in the group who did not work from home. Similarly, the coefficient β_4 indicate the differential change from baseline to the AfterRestrict period among those working from home, relative to the change in the group who did not work from home. The main effect of WorkedFromHome is redundant due to the fixed effects (Individual_i) that account for between-person differences. Calculation of standard errors were cluster-robust and allowed for correlated errors within individuals.

Supplementary Text S2. Supplementary Analyses

To explore potential non-linear effects of the number of days working from home, we substituted the linear number of days working in the first model with dummy variables for each of the response categories (one day per week, two, three, four, and five days or more) with “no days” as the reference category. The regression predicting Drinking Frequency did not suggest a dose-response effect of the number of days working from home according to the following coefficients ordered from one to five days working from home (standard errors in parentheses): 0.38 (0.21), 0.11 (0.39), 0.33 (0.40), -0.56 (0.51), 0.50 (0.37). Similarly, results with HED as the outcome did not suggest an increase in the frequency of heavy drinking episodes with increasing number of days working from home: 0.24 (0.25), 0.55 (0.42), -0.29 (0.46), 0.48 (0.44), 0.10 (0.39). All p-values > .17.

To explore whether heavy drinkers were more vulnerable to the effects of working from home we calculated the average frequency of HED occasions in the pre-pandemic period using an extended dataset from 2015-2019. In an analysis of employees with pre-pandemic HED scores from the 95th percentile and above, who also participated in 2020-2023 (Individuals=41, observations=91), we observed a tendency for remote work to be associated with an increase in Drinking Frequency, B=1.11 (95% CI -0.41−2.64), but this tendency was not statistically significant, $p = 0.15$. The coefficient was also in the same direction for the dependent variable HED but not statistically significant (Individuals=38, observations=85), B=0.30 (95% CI -0.31−0.90), p = 0.33. When using a lower cut-off, the 80th percentile, for classifying pre-pandemic heavy drinkers, the coefficients were attenuated, B=-0.01 (95% CI -0.26−0.24) for Drinking Frequency (Individuals=143, Observations=329) and B=0.14 (95% CI -0.63−0.92) for HED (Individuals=146, Observations=336). If working from home affects the drinking among the group of employees who drink the most, the above analyses indicate that we do not have the statistical power to detect such an effect.
